# Erosive effect of industrialized fruit juices exposure in enamel and dentine substrates: An *in vitro* study 

**DOI:** 10.4317/jced.57385

**Published:** 2021-01-01

**Authors:** Ingrid-Andrade Meira, Elis-Janaina-Lira dos Santos, Nayanna-Lana-Soares Fernandes, Emerson-Tavares de Sousa, Andressa-Feitosa-Bezerra de Oliveira, Fábio-Correia Sampaio

**Affiliations:** 1 Department of Prosthodontics and Periodontology, Piracicaba Dental School, University of Campinas-UNICAMP, Piracicaba/São Paulo, Brazil; 2 Department of Clinical and Social Dentistry, Federal University of Paraiba-UFPB, João Pessoa/Paraiba, Brazil; 3 Department of Health Sciences and Pediatric Dentistry, Piracicaba Dental School, University of Campinas-UNICAMP, Piracicaba/São Paulo, Brazil; 4 Department of Morphology, Federal University of Paraiba-UFPB, João Pessoa/Paraiba, Brazil; 5 Department of Clinic and Social Dentistry, Federal University of Paraiba-UFPB, João Pessoa/Paraiba, Brazil

## Abstract

**Background:**

Erosive tooth wear has been a highly prevalent and emerging phenomenon related to eating habits of the population. Aim: This study sought to investigate industrialized fruit juices exposure in enamel and dentine substrates in terms of erosive effect.

**Material and Methods:**

Human enamel and dentine specimens were randomized into 8 groups (n=8): Grape juice – Ades®, Grape juice – Del Valle Kapo®, Grape juice – Aurora®, Orange juice – Del Valle Kapo®, Orange juice – Ades®, Strawberry juice – Mais Vita®, Strawberry juice – Ades®, Citrus fruit juice – Tampico®. Specimens were submitted to an in vitro erosive challenge and to a microhardness test to evaluate the percentage of surface microhardness loss. The pH, titratable acidity, buffering capacity, degree of saturation and critical pH concerning hydroxyapatite and fluorapatite of the juices were measured as well as their composition of calcium, phosphate, fluoride, and total protein. Data were submitted to the analysis of variance and multivariate linear regression (α=0.05).

**Results:**

All test agents were undersaturated concerning hydroxyapatite and fluorapatite. A significant interaction between the type of juice and substrate was found (α=0.000, β=0.99). However, Orange juice – Del Valle Kapo®, Orange juice – Ades®, and Strawberry juice – Mais Vitta® demonstrated no difference between substrates. Grape juice – Ades® promoted less mineral than other juices in enamel and dentine. The calcium concentration in juices was a protective variable for microhardness loss in both substrates.

**Conclusions:**

The erosive effect of industrialized fruit juices affects enamel differently from dentine, and this effect differed between some, but not all, tested juices.

** Key words:**Tooth erosion, dental enamel, dentine. beverages, food habits.

## Introduction

Lifestyle changes in the past few decades, mainly those related to the patters of food consumption (high intake of acidic drinks and food), call the attention of the global scientific community for erosive tooth wear (ETW) as highly prevalent and emergent phenomena (1-3). Epidemiological studies that investigated the influence of diet and ETW prevalence evidenced that some dietary components as carbonated drinks (OR 1.61, 95% CI 1.29–2.01) and natural acidic fruits juice (OR 1.20, 95% CI 0.02–1.41) increased erosion occurrence (3).

The erosive lesions caused by acidic foods and beverages were related to their physicochemical properties as well as the concentration of acids, power of hydrogen, titratable acidity, buffering capacity, inorganic concentration of ions calcium, phosphate, fluoride, and mineral chelating properties. Therefore, a potentially erosive agent should have reduced ionic activities of Ca2+, PO43–, and F- and negative saturation degree concerning hydroxyapatite (HAp) and fluorapatite (FAp) crystals (4,5). Besides, the concentration of proteins could influence the erosive effect of beverages, probably because of the protein buffering effect under acidic circumstances (6-8). One example of this, it is the casein protein supplementation, which has reduced the erosive potential of acid fruit juices (8,9) and citric acid solutions (6,10).

The erosive process in the enamel and dentine substrates is different due to their different histopathological aspects. While enamel erosion was expressed as superficial demineralization followed by softening and substance loss, dentine erosion beginning with a mineral loss between peritubular and intertubular dentine with subsequent peritubular dentine loss and tubule orifice enlargement. Besides, differences in organic and water components play an important role in the diffusion of H+ ions, which could determine a different behavior when exposed to erosive agents (4,11).

Dentine erosion was particularly evident in adults with physiological or pathological root exposure, and the more relevant clinical consequence could be dentine hypersensitivity and an increase in biofilm stagnation. Prevalence data are scarce in adults individuals, even though data from epidemiological studies evidenced that erosive lesions tend to increase and became more severe during the aging process (12). Given this context, the aim of this *in vitro* study was to evaluate the erosive effect of commonly available industrialized fruit juices in enamel and dentine substrates.

## Material and Methods

-Ethical Consideration

Considering the use of human teeth, a Research Ethics Committee in Brazil approved this study (CAAE: 17581413.4.0000.5188). The teeth donors were explained about the aims and relevance of this research and signed the Informed Consent Form according to the Helsinki Declaration and the Brazilian resolution (CNS/MS 466/12).

-Experimental Design

This research was conducted with human enamel and dentine samples in three stages:

Stage 1: The selected juices were evaluated in terms of pH, titraTable acidity (TA), buffering capacity (β) and the amount of calcium [Ca2+], inorganic phosphate [Pi], fluoride [F-], total protein composition, and degree of saturation (pK − pI) and critical pH concerning hydroxyapatite (HAp) and fluorapatite (FAp).

Stage 2: Microhardness test to evaluate the percentage of surface microhardness loss (%SMHL) was performed in human enamel (N=64) and dentine (N=64) blocks. These specimens were randomly assigned into the following eight (8) fruit juices groups, each one with 8 specimens: (1) Grape juice – Ades®, (2) Grape juice – Del Valle Kapo®, (3) Grape juice – Aurora®, (4) Orange juice – Del Valle Kapo®, (5) Orange juice – Ades®, (6) Strawberry juice – Mais Vita®, (7) Strawberry juice – Ades®, (8) Citrus fruit juice – Tampico®, and positive control with Coca-cola – Coca Cola®.

Stage 3: Erosive challenge and subsequent microhardness test to evaluate the %SMHL.

-Beverages

Ready-to-drink fruit juices, potentially erosive and frequently consumed, identified in Brazilian supermarkets were selected according to the flavor and manufacturing. They were kept closed at room temperature until the day of analysis and experiment. The composition, brand, and manufacturer of then are present in Box 1.

-Physicochemical Characterization of the Beverages

Initially, the selected beverages had their pH measured shortly afterward the opening by means an electrode connected to a pH meter (Orion Model 420A -Thermo Fischer Science Inc., Waltham, MA). In 50 ml of beverages was added 0.5 ml of 1 M NaOH until the pH of 5.5 and 7.0, under constant agitation, to calculate the titratable acidity (TA). It was used the Equation: β=∆C/∆pH to calculate the buffering capacity (β), where ΔC is the quantity of base used and ΔpH is the alteration in pH caused by the addition of the base (5). The measurements were made in triplicate.

In the next stage, it was used the direct colorimetric method by microplate spectrophotometer (PowerWave HT, BioTek Instruments, Winooski, VT, USA) for measurements of calcium and phosphate concentrations. Briefly, it was pipetted 25 µL of each beverage in a 96-well plate to react with a calcium-sensitive reagent (Arsenazo III) and reducing phosphate agents (molybdic acid and alpha-aminonaphthol sulfonic acid). The reader was pre-calibrated with a standard curve of calcium carbonate (0- 100 mg/L) and phosphate (0-8.27 mg/L). The readings were performed using the absorbance of λ 650 nm for calcium and the absorbance of λ 660 nm for phosphate (13). Using a linear equation (y = ax + b) at a curve fit above 0.98, the ionic concentration was calculated from the absorbance values. Samples, in triplicates, were analyzed and the variation coefficient of the calcium and phosphate analysis was lower than 5%.

The fluoride analysis was performed using the microdiffusion method (14). A fluoride-ion-specific electrode (BN Model 9409, Orion, Cambridge, MA, USA) and a potentiometer (Model 720 Orion, Cambridge, MA, USA) were used to reach this analysis, and each sample was analyzed in triplicates. Before readings, the electrode calibration was performed with fluoride standards of 0.02 to 2.56 μg of F/mL, under the same conditions as samples. Standard samples were prepared by dilution of a stock solution containing 0.1 M F (Orion 940906). The millivolt readings were converted to fluoride ion concentration using a standard correlation curve (r²> 0.99) based on linear equation (y = ax + b).

Calcium, phosphate, and fluoride concentrations were shown as mmol/L.

The Bradford colorimetric method (15) using Bradford’s reagent (Sigma Aldrich, Jurubatuba-SP, Brazil) was used to define the protein concentration. A calibration curve of standard concentrations (0.01 – 0.08 μg/μL) of bovine serum albumin (Sigma Aldrich, Jurubatuba-SP, Brazil) was used to reach the conversion of absorbance values to μg/μL of protein. To sum up, in 96-well plates containing 200 μL of Bradford reagent were included 10 μL of samples. After this, this content was incubated at 22°C for 5 minutes and analyzed using a microplate spectrophotometer (PowerWave HT, BioTek Instruments, Winooski, VT, USA) at λ 595 nm of absorbance. Results were expressed as µg/mL.

Degree of Saturation and Critical pH of Beverages

**Figure 1:**
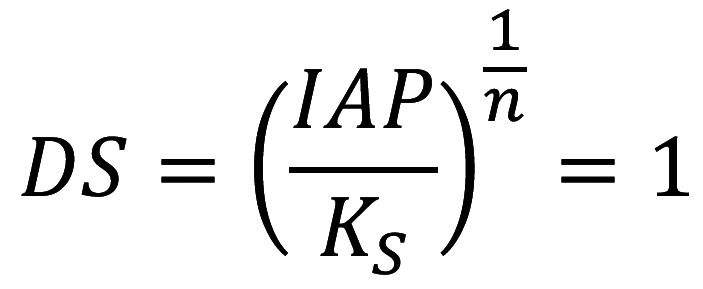
Formula.

A computer program (16) was used to calculate the degree of saturation (pK-pI) and the critical concerning hydroxyapatite (HAp) and fluorapatite (FAp). These calculations were performed using pH and the concentrations of Ca2+, *Pi*, and F- values of each juice and assuming a solubility product of 10-58•5 and 10-59•6 for HAp and FAp, respectively. Alternatively, the critical pH was assessed through the simulation of the degree of saturation concerning HAp and FAp at several pH values. Thus, the pH value at which the solution is exactly saturated concerning HAp and FAp (Fig. 1), is considered as the critical pH of the solution.

-Sample Size and Sample Preparation

**Figure 2:**
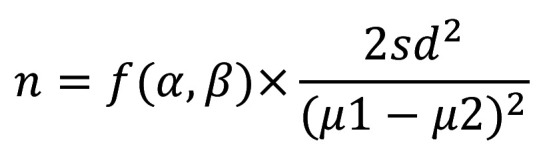
Formula.

Based on the anterior study (17), the sample size calculation was carried out to compare the least difference in enamel mineral loss between soy-based beverages of different flavors. The number of enamel samples necessary in each group is given by the following formula (18): (Fig. 2).

Where μ1-μ2 is the smallest difference in means between Grape (0.5) and Orange (0.7) juices, f(α,β) is a function of α (0.05) and β (0.05), sd is the standard deviation of the Grape Juice (0.1). In each group, the final sample size was 6.5 enamel blocks and it was raised by 20% for offset possible losses during the experiment. Thus, 8 samples in each experimental group were necessaries.

One hundred twenty-eight human third molars were collected and these teeth were equally divided into enamel and dentine experiments. Initially, they were cleaned through scraping with periodontal curettes and stored in buffered 10% formalin (pH 7.0) solution. After this, each teeth surface was examined through a magnifying glass with an increase of 5x to ensure that the selected tooth did not have caries, cracks, intrinsic staining, and none malformation in the enamel and dentine tissue.

After the teeth selection, a double-sided disk of the precision diamond cutter (Labcut 1010- Extec Corp, London, England), under slow-speed and constant irrigation, was used. Enamel and dentine blocks (3x3x2 mm) were obtained from the dental crown (buccal and lingual surfaces). The specimens were embedded in self-cured acrylic resin circular using molds of 16mm diameter and 3mm deep. Later, a metallographic polisher was used to ground flat the outer enamel (sandpaper disks with granules 400, 600, 1200) and dentine (sandpaper disks with granules 600, 1200) surfaces, under constant irrigation. The polishing enamel and dentine surfaces were performed with wet felts and 1 µm diamond suspension (Extec Corporation, Enfield, CT) in a rotating polishing Machine PSK- 2V (Skill-tec Comércio e Manutenção Ltda, São Paulo, SP, Brazil). Finally, all samples were included in a sonication with water for 5 min and stored under -20ºC until the day of the experiment.

-Erosive Challenge

The enamel and dentine samples were immersed into 50 ml (5.56 ml of the fruit juice/ mm2 exposed enamel or dentine specimens), at room temperature (22-25ºC), for 120 minutes (19), under gentle agitation. At the end of the erosive challenge, the specimens were washed again with deionized water under sonication for 5 min and stored in a humidity-controlled environment to prevent drying until further analysis.

-Analyses of Microhardness 

**Figure 3:**
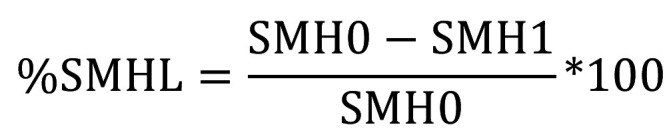
Equation.

A microhardness machine (Shimadzu HMV-AD Micro Hardness Tester, Japan) determined the surface hardness of the enamel and dentine specimens before and after the erosive challenge. For this, a Vickers diamond with 100g for 15 seconds was used for the enamel specimens and 50g for 10 seconds for dentine specimens. Initially, in the dentine and enamel samples sound surface, three indentations were performed with an interval of 70 µm from one indentation to another. The dimension of each indentation was used for WIN-HCU software to calculate the hardness of the tissues. After the erosive challenge, the same procedure was performed in the eroded area. Finally, the %SMHL was calculated using the following equation: (Fig. 3).

Where the SMH0 is the average microhardness of the sound tissues and SMH1 is the average microhardness of the eroded tissues.

-Statistical Approach 

Data were statistically analyzed using the SPSS package for Windows, version 21.0 (SPSS, Inc., Chicago, IL, USA). Data normality and homogeneity of variances were tested using the Shapiro-Wilk test and Levine test, respectively. Since data demonstrated homogeneity of variances and Gaussian distribution, no data transformation was needed.

Assumptions of analysis of variance were checked before the use of two-way factorial analysis of variance (ANOVA) model to investigate simple and interaction effects of two factors (Juice and Substrate) among the dependent variable (%SMHL). Levine’s test was used to prove the equality of multiple variance-covariance matrices considering the level of significance of 0.05 (*p*-value of Levene’s test of equality of error variances was equal to 0.128). Alpha (α), beta (β), and partial eta squared (ηp²) represent, respectively, the type I error, effect size, and the strength of each the effect. Simple effects were used to assess the difference between the substrate within each juice. Multiple comparisons between juices within each substrate were performed by Fisher’s Least. Significant difference test F followed by Bonferroni correction as a post hoc analysis of ANOVA.

The curve estimation procedure was used to graphically screen data and to prove that independent and dependent variables were linearly related. Multiple linear regression with adjusted r² value and 5% of significance. Independent variables were included in the model based on the backward elimination procedure, which considers the removal of independent variables to find the most statistically significant improvement of the model. Two explanation models were made to verify the relative influence of each independent variable on the %SMHL according to the dental substrate – Enamel (Model 1) and Dentine (Model 2). Collinearity tests considered tolerance above 0.1 and the variance inflation factor (VIF) lower than 10. Variables as TA, critical pH and degree of saturation were not included in the model due to their high interdependence with the other variables. The supposition of error independence was tested using the Durbin-Watson statistic.

## Results

Table 1 summarized that all tested beverages had initial pH values below 5 (2.94 to 4.01) and were undersaturated concerning the HAp and FAp. All test agents under study were undersaturated concerning both HAp and FAp. The larger titraTable acidity and buffering capacity were found for Grape juice – Aurora®, Orange juice – Del Valle Kapo®, and Citrus fruit juice – Tampico®. The highest concentration of calcium was found in Grape juice – Ades® and Orange juice – Ades® whereas the concentration of phosphate, fluoride, and total protein were highly variable among juices.

**Table 1:**
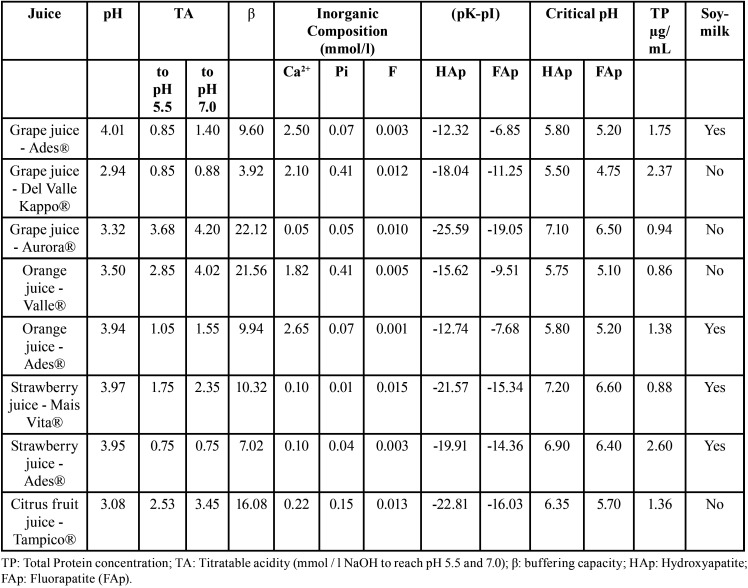
Physicochemical parameters of each tested juice.

**Figure 4:**
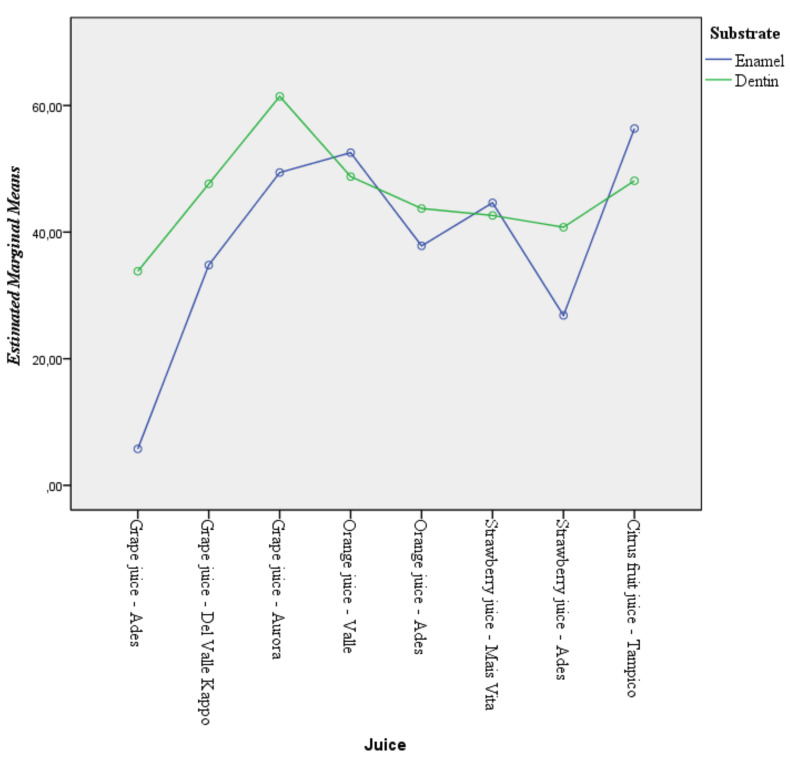
Evaluation of the interaction between juices treatment and the substrate on the %SMHL.

The type of juice affects the %SMHL in enamel differently from dentine (significant interaction effect); however, the %SMHL after challenge with Orange juice – Del Valle Kapo®, Orange juice – Ades®, and Strawberry juice – Mais Vitta® did not differ between substrates (Table 2). The profile plot was provided in Figure 4 to represent the interaction between substrate and juices.

**Table 2:**
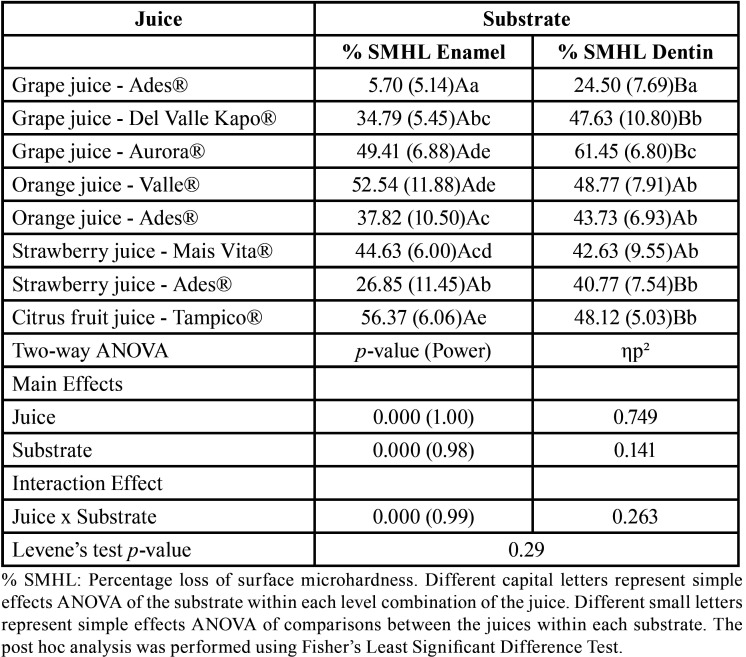
Main and interaction effect between the erosive challenger of each juice and the substrate on the percentage loss of surface microhardness: a two-way analysis of variance.

Simple effects (analysis of juice effect individualized by substrate) analysis demonstrated that the influence of the juices was significant in enamel and dentine substrates. The high effect size (Type II error) and partial eta squared of these influences evidence that these effects and their interaction are strong and with minimal likelihood of error. Post hoc analysis between juices within each substrate demonstrated that Grape juice – Ades® promoted less mineral loss than other juices in both substrates. A higher percentage of SMHL was found in Grape juice – Aurora®, Orange juice – Del Valle Kapo®, and Citrus fruit juice – Tampico® in enamel, and Grape juice – Aurora® in dentine when compared with other juices (Table 2).

Table 3 demonstrated that the calcium concentration in juices was a protective variable for %SMHL in enamel and dentine. The increase in the juice pH was a protective factor for %SMHL in dentine (Model 2). Besides, the protective effect of fluoride was only relevant in Model 2.

**Table 3:**
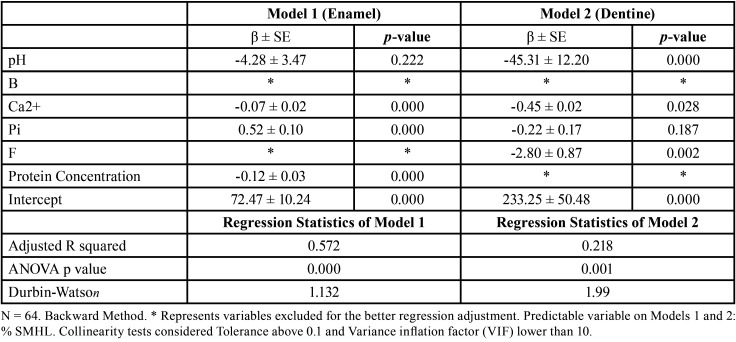
Explanation models for the % SMHL on the enamel and dentin surface.

## Discussion

It is noticeable the importance of screening the erosive potential of drinks and foods to obtain evidence-based information for a more comprehensive diet counseling for patients with risk of dental erosion. The consumption of carbonated drinks and fruits juice has increased erosion occurrence, while whole milk and yogurt have presented a protective effect (20). This is particularly important when it is considered that some type of fruit juices could be as erosive as soft drinks (5,7,17).

Ready-to-drink juices that mimic fresh juices contain citric acid and other low-pH preservatives that could be an extrinsic source for ETW. The chemical behavior of citric acid has been dependent on two modes of actions: one related to their acid dissociation constant (pKa) and another to their chelating ability. Thus, formulations with this acid are very damaging to the tooth surface. Double chemical action is also found in phosphoric acid (a component of Coca-Cola), which has higher ionic strength and calcium affinity than citric acid (21). For this reason, this experiment considered Coca-Cola - Coca-Cola® as our positive control. Nevertheless, the majority of the tested fruit juices had more erosive effects than Coca-Cola – Coca-Cola®.

Despite biomechanical and histologic differences between enamel and dentine substrates, studies comparing the dental erosion effect on these biological tissues are scarce. Accordingly, this study was designed to provide an overview regarding the effect of potentially erosive commercially available fruit juices on enamel and dentine in terms of %SMHL. The interpretation of this research should consider that, under the circumstances of an *in vitro* single-exposure erosion simulation, this study does not provide information about what happens in the oral cavity since behavioral and biological host-related factors have a strong influence on the ETW pathogenesis (11).

The main results revealed that changes in the erosive behavior of fruit juices can be found according to the type of substrate and type of fruit juice. Mineral loss in dentine tends to be higher than enamel, which could be expected since dentine has lower mineral (almost 47 vol%) (21), and higher solubility (22). However, at a high time exposure, it was demonstrated that the presence of an organic matrix on the dentine substrate could have a protective effect on substance loss caused by acidic beverages when compared with enamel substrate (23). Conflicting results with the study carried out by Zimmer *et al.* (23) could be due to experimental design, methods of assessing erosion and type of acid treatment.

The relative influence of ionic strength of calcium on the erosive potential of juices proved to be important on enamel and dentine specimens (Table 3), probably because of the greater need for calcium ions surrounding tooth for creating a more saturated environment concerning the HAp and FAp minerals when compared with fluoride and phosphate ions. The less erosive effect of calcium-fortified fruit juice was previously demonstrated in others an *in vitro* studies (24,25). Besides, it is possible that the stabilization of calcium phosphate in solution as observed in some products with casein phosphopeptide-amorphous calcium phosphate (CCP-ACP) could be particularly important (26).

An important distinction regarding the acid demineralization in dental caries and dental erosion is that there is no established critical pH concerning HAp and FAp crystals. Thus, little variations on chemical composition and physicochemical properties of juices could provide a relevant modification on the saturation degree of dental minerals and a different pattern of mineral loss and tissue damage (27). In the present study, the critical pH concerning HAp and FAp ranged from 5.5 to 7.2 and 5.1 to 6.6, respectively. Higher values of critical pH were found in Grape juice – Aurora® and Strawberry juice – Mais Vita®. However, the effect Grape juice – Aurora® on the %SMHL was higher than Strawberry juice – Mais Vita®. Probably, lower values of TA and buffering capacity (almost 50% lower) associated with the presence of soymilk in Strawberry juice – Mais Vita® provides important protection against more severe damage to the enamel structures. The relative influence of soymilk on dental erosion was evidenced in a previous study (7).

Regarding the significant protective effect of fluoride on the dentine substrate, our data suggest that fluoride even in low concentration could reduce the erosive damage of juices. On the other hand, in enamel specimens, the concentration of fluoride is not enough to detect a protective effect. The limited effect of fluoride on erosive lesions have been previously reported (24,28).

Interestingly, pH was only relevant for dentine substrate. The multiple and linear regression model evidenced that if the juice pH is increased by one unit, a reduction of 45.31 ± 12.20 in the SMHL could be predicted. It is possible that the amount of H+ freely available influences the buffering properties of collagen and other proteins found in dentine, which is dependent on their isoelectric point (the tridimensional structure has to be charged to exert buffer effect), as reported by Lussi *et al.* (11). In addition, the absence of a significant effect of protein concentration in the juices on the dentine %SMHL (Table 3) could be a sign that the dentin protein matrix overcomes the protective effect of proteins in juices. Deeply understanding of these hypotheses and their implications on the complex histology of erosive lesions in dentin could be an interesting topic for future researches.

Therefore, the erosive effect of industrialized fruit juices affects enamel differently from dentine, and this effect differed between some, but not all, tested juices. Besides, differences in the physicochemical properties (pH for dentin) and inorganic composition of juices (Ca2+ and *Pi* for enamel and Ca2+ and F- for dentin) could explain their different erosive behavior effect on both substrates.
